# Supplementary material for: Topology selectivity of a conformationally flexible precursor through selenium doping
Source: Nat Commun. 2024 Apr 15;15:3235. doi: 10.1038/s41467-024-47614-9 (PMC11018763; doi:10.1038/s41467-024-47614-9)
Supplement: Supplementary file 1 — Supplementary Information [file 41467_2024_47614_MOESM1_ESM.pdf]

Supplementary Information

Topology Selectivity of a Conformationally Flexible Precursor through Selenium Doping

Liangliang Cai,[a] Tianhao Gao,[a] and Andrew T. S. Wee[a]*

[a] Department of Physics, National University of Singapore, 2 Science Drive 3, Singapore 117542

Table of content

Supplementary Figure 1. Random phases after deposition of mTBPT on Cu(111).

Supplementary Figure 2. Crystallization from random phase at RT through Se doping on Cu(111).

Supplementary Figure 3. STM images with different ratios of Se to mTBPT after thermal treatment (365 K for 2 h).

Supplementary Figure 4. Electronic properties of the nanopores.

Supplementary Figure 5. DFT calculated electronic properties of the ordered organometallic network in gas phase.

Supplementary Figure 6. Periphery interactions C−Cu…Se of the network.

Supplementary Figure 7. The IRI map of finite C−Cu…Se moiety.

Supplementary Figure 8. The energy barrier for cleaving the Cu…Se interaction.

Supplementary Figure 9. Deposition of Se on cold Cu(111) pre-covered with random OMs.

Supplementary Figure 10. Energy barriers for sliding diffusion of the C_s−Cu moiety.

Supplementary Figure 11. Energy barriers for sliding diffusion of the C_{3h}−Cu moiety.

Supplementary Figure 12. Energy barriers for rotations barriers of C_s−Cu and C_{3h}−Cu moieties, respectively.

Supplementary Figure 13. Scheme of mTBPT reaction pathway.

Supplementary Figure 14. STM images after further annealing from random (without Se doping) or ordered (with Se doping) organometallics.

Supplementary Figure 15. DFT calculations on the covalent interlinked 1D chain and 2D network.

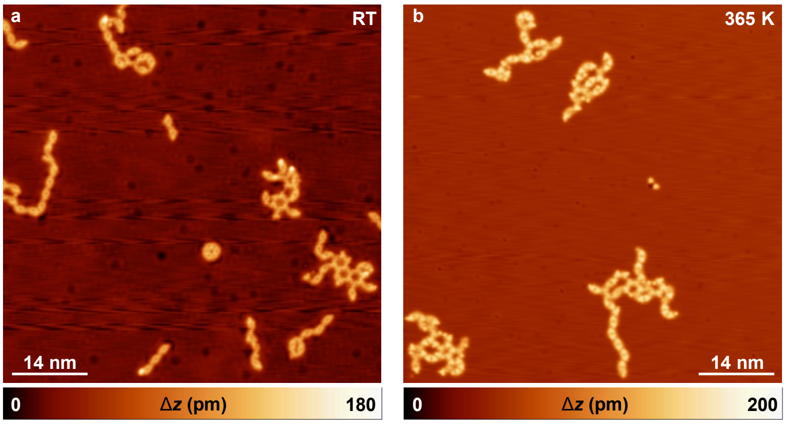

Supplementary Figure 1. **Random phases after deposition of mTBPT on Cu(111).** STM images showing the random phase formed after deposition of *m*TBPT on Cu(111) at (a) RT and (b) 365 K. STM parameters: constant current, (a) U = -1 V, I = 100 pA; (b) U = 1 V, I = 100 pA.

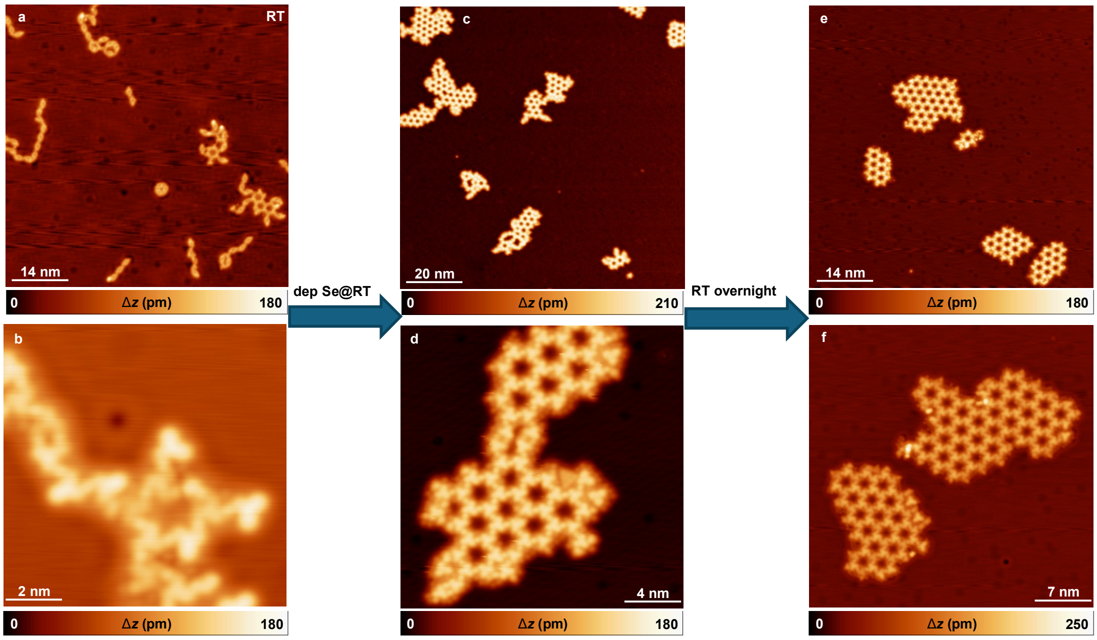

Supplementary Figure 2. Crystallization from random phase at RT through Se doping on Cu(111). (a) Large-scale and (b) close-up STM images showing the random phase formed by *m*TBPT on Cu(111) at RT. (c) Large-scale and (d) close-up STM images indicating the transformation from the random phase to 2D ordered crystalline phase after deposition of Se atoms, still with some non-hexagonal pores. (e) Large-scale and (f) close-up STM images showing ordered 2D MOFs with few non-hexagonal pores after annealing at RT overnight (for 12 h). STM parameters: constant current, (a) U = -1 V, I = 100 pA; (b) U = 500 mV, I = 100 pA; (c) U = 2 V, I = 10 pA; (d) U = 2 V, I = 1 nA; (e, f) U = 1 V, I = 100 pA.

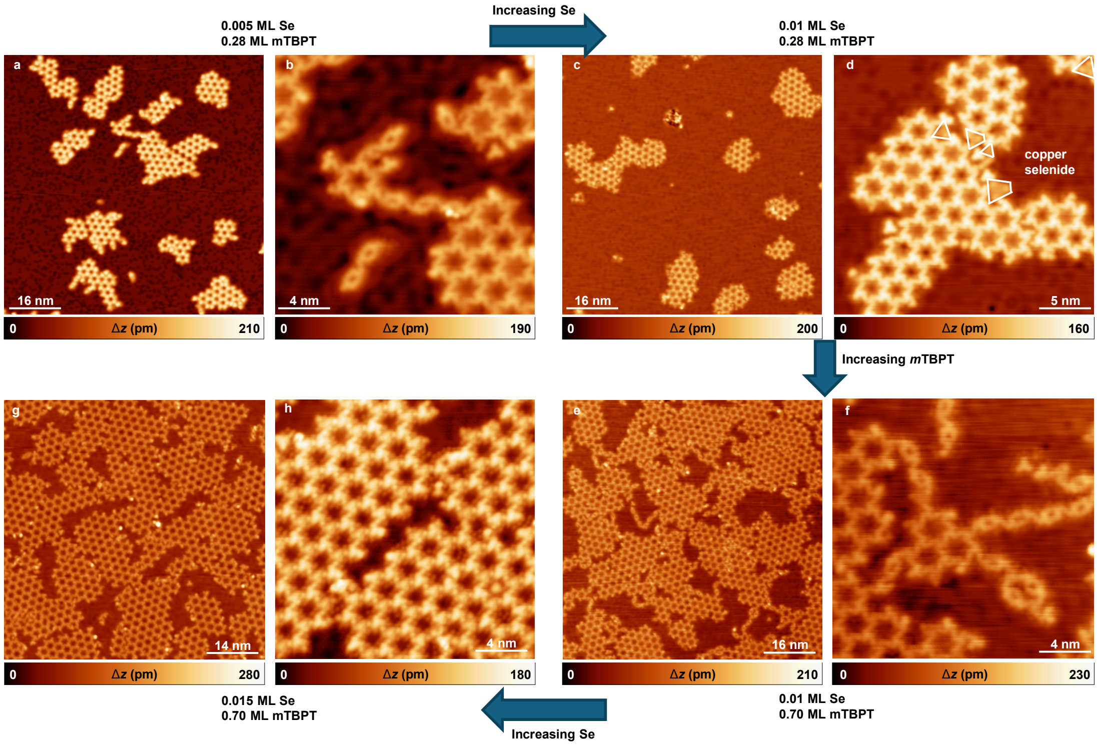

Supplementary Figure 3. STM images with different ratios of Se to *m*TBPT after thermal treatment (365 K for 2 h). (a) Large-scale and (b) close-up STM images showing coexistence of 1D chains and 2D networks after co-deposition of 0.005 ML Se and 0.28 ML *m*TBPT. (c) Large-scale and (d) close-up STM images showing 2D networks and small copper selenide domains after increasing the coverage of Se (0.01 ML Se and 0.28 ML *m*TBPT). (e) Large-scale and (f) close-up STM images showing 2D networks (growing bigger) and some 1D chains forming after further increasing the coverage of *m*TBPT (0.01 ML Se and 0.70 ML *m*TBPT). (g) Large-scale and (h) close-up STM images showing 2D networks with negligible 1D chains after adding more Se (0.015 ML Se and 0.70 ML *m*TBPT). STM parameters: constant current, (a, g, h) $U = 1$ V, $I = 50$ pA; (b) $U = 200$ mV, $I = 100$ pA; (c, e, f) $U = 500$ mV, $I = 50$ pA; (d) $U = 400$ mV, $I = 100$ pA.

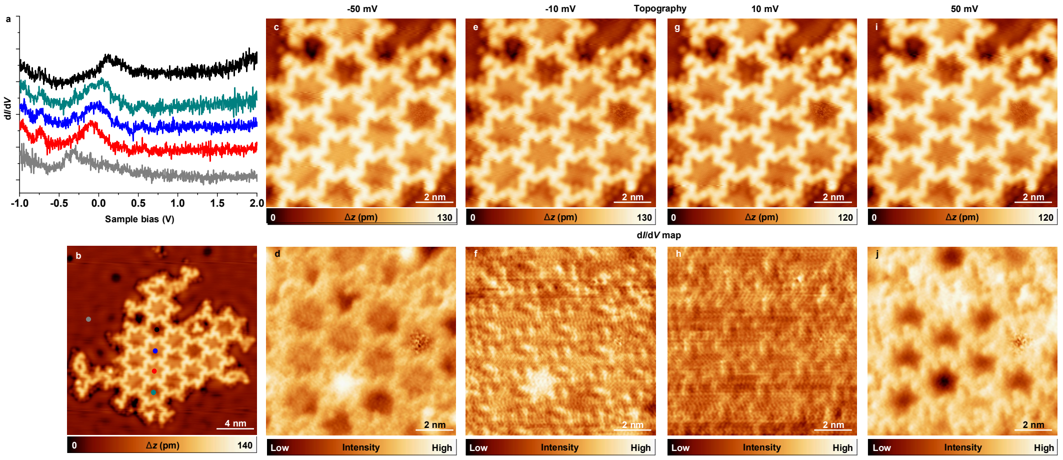

Supplementary Figure 4. Electronic properties of the nanopores. (a) d*I*/d*V* spectra (set point:

−1 V, 1 nA) measured at the center of pores with different numbers of residual bromine atoms as indicated in panel b (red, 0; blue, 1; green, 2; black, 3), and revealed the confined surface state of Cu(111). The spectra are vertically shifted for clarity. STM topographic image and the corresponding d*I*/d*V* mapping recorded at (c, d) -50 mV, (e, f) -10 mV, (g, h) 10 mV, (i, j) 50 mV. The d*I*/d*V* spectra were acquired by a lock-in amplifier while the sample bias was modulated by a 963 Hz, 20 mV (r.m.s.) sinusoidal signal under open-feedback conditions. Tunneling parameters: (b) U = -100 mV, I = 1 nA; (c, e, g, i) I = 1 nA. The bias was applied to the sample. STM images were recorded in a constant-current mode. Source data are provided as a Source data file.

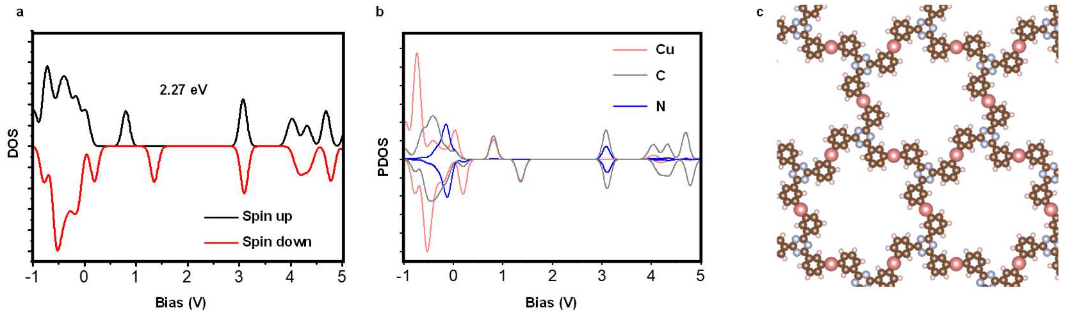

Supplementary Figure 5. DFT calculated electronic properties of the ordered organometallic network in gas phase. (a) Spin-polarized density of states, (b) projected density of state, and (c) the corresponding DFT model of the ordered organometallic network in gas phase. Black and red lines in (a) represent the spin-up and spin-down contributions, respectively. Pink, gray and blue in (b) represent the Cu, C and N contributions, respectively. Source data are provided as a Source data file.

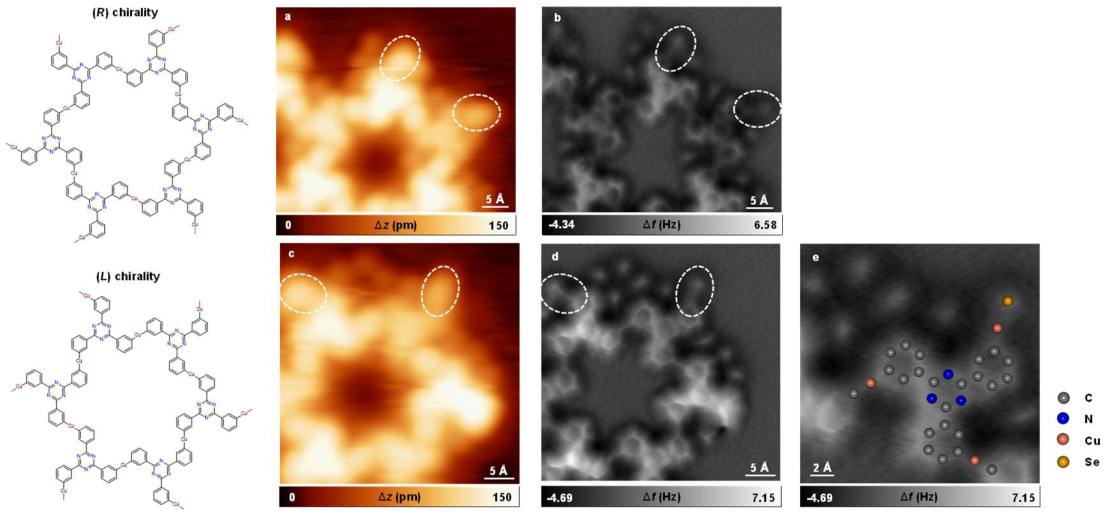

Supplementary Figure 6. Periphery interactions C−Cu…Se of the network. (a) The current image and (b) corresponding nc-AFM frequency shift image of (*R*)-network acquired at the same position show the periphery interactions. (c) The current image and (d) corresponding nc-AFM frequency shift image of (*L*)-network acquired at the same position show the periphery interactions. White ellipses indicate the terminal interactions to the periphery molecules. (e) High-resolution of nc-AFM frequency shift image superimposed with the model indicates the C−Cu…Se interaction of the network periphery. Scanning parameters: (a, c) constant current mode with U = 1 V, I = 100

pA. (b, d, e) constant height mode using a CO-terminated tip at a bias voltage of 0 V, images were recorded at the tip offset $\Delta z = -250$ pm with respect to an STM set point ($U = 1$ V, $I = 100$ pA). Source data are provided as a Source data file.

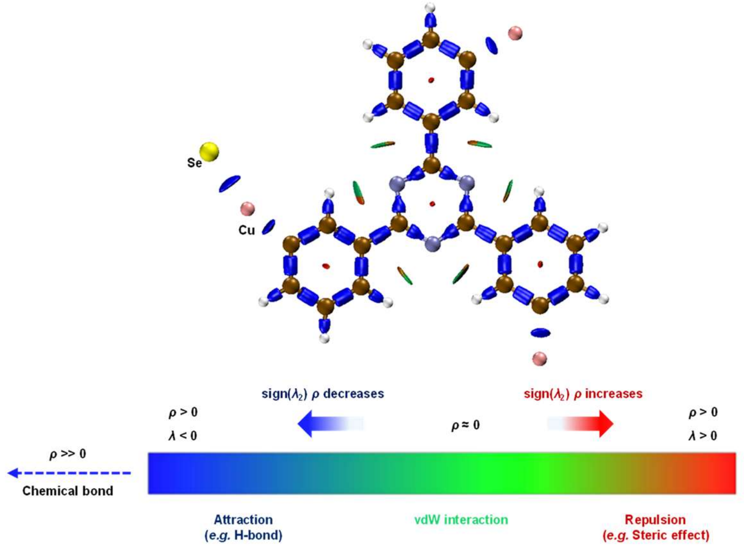

Supplementary Figure 7. The IRI map of finite C−Cu…Se moiety. Upper: The IRI isosurface map of finite C−Cu…Se moiety showing the interactions between the Se adatom and the C−Cu species. Blue and green isosurfaces represent the covalent interactions and weak interactions, respectively. Lower: Standard coloring method. Brown, white, gray, pink, and yellow balls represent C, H, N, Cu, and Se atoms, respectively. The scales of color bars are given in a.u. Source data are provided as a Source data file.

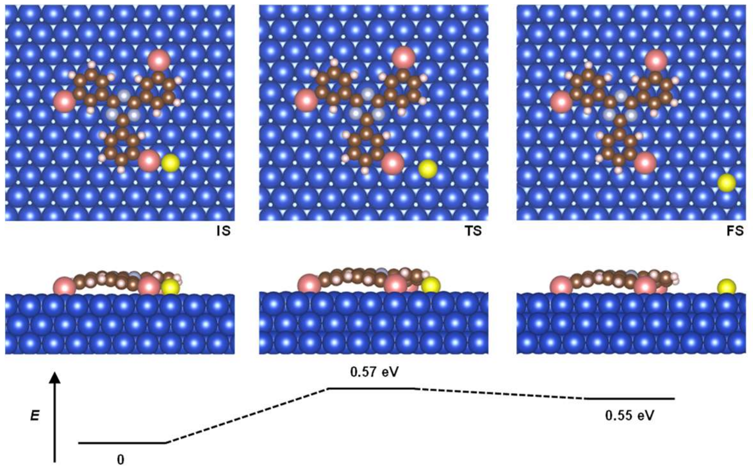

Supplementary Figure 8. **The energy barrier for cleaving the Cu…Se interaction.** DFT-calculated energy diagram for breaking the interaction between C−Cu and the Se atom in the C−Cu…Se terminal. The structural models are given for the initial, transition and final states, respectively. Brown, white, gray, yellow, blue, and pink balls represent C, H, N, Se, Cu substrate, and Cu adatoms, respectively. Source data are provided as a Source data file.

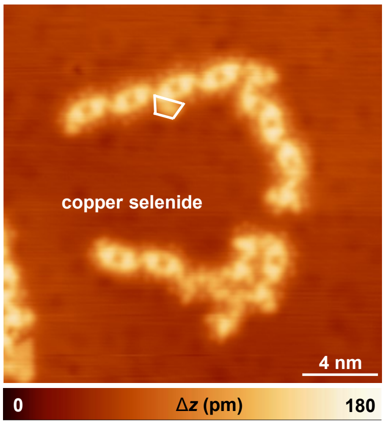

Supplementary Figure 9. **Deposition of Se on cold Cu(111) pre-covered with random OMs.** The STM image showing deposition of Se at low temperature (≈ 200 K) on the Cu(111) surface pre-covered with random organometallics. The copper selenide outlined by white trapezoid is located beside a C_s–Cu moiety. STM parameters: constant current, U = 1 V, I = 100 pA.

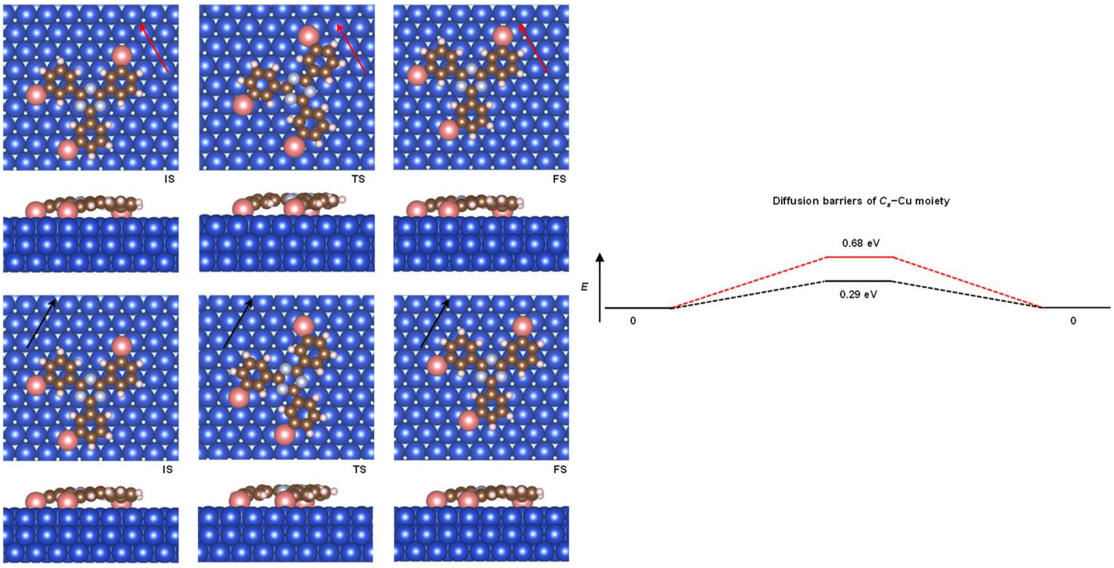

Supplementary Figure 10. Energy barriers for sliding diffusion of the C_s–Cu moiety. DFT-calculated energy diagrams for sliding diffusion of the C_s–Cu moiety on Cu(111), where the top and side views of the paths are depicted in the left panel for two diffusion directions (indicated by red and black arrows respectively). The structural models are given for the initial, transition and final states, respectively. Brown, white, gray, blue, and pink balls represent C, H, N, Cu substrate, and Cu adatoms, respectively. Source data are provided as a Source data file.

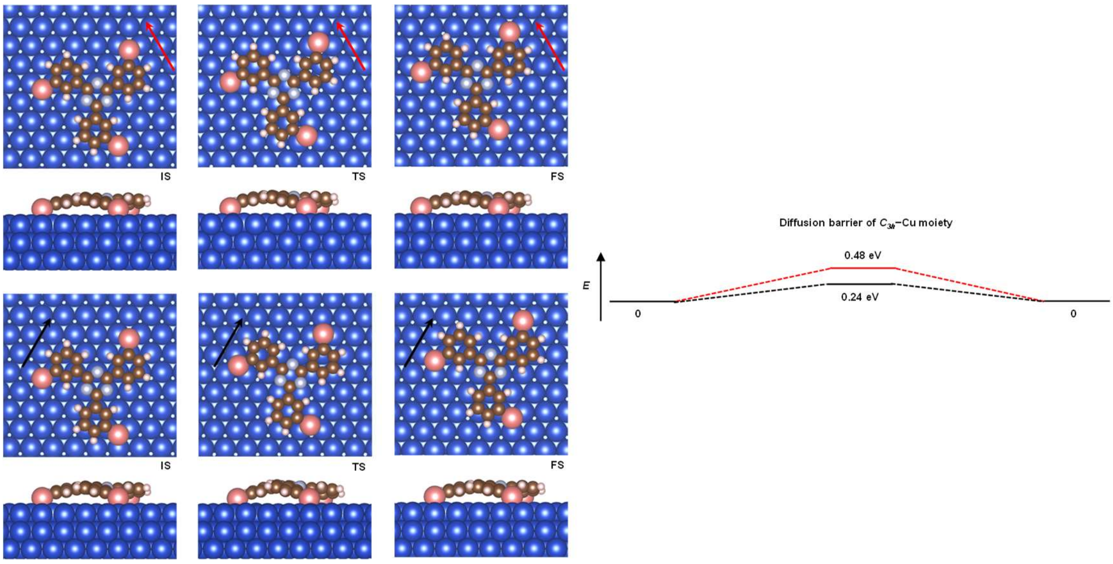

Supplementary Figure 11. Energy barriers for sliding diffusion of the C_{3h}−Cu moiety. DFT-calculated energy diagrams for sliding diffusion of the C_{3h}−Cu moiety on Cu(111), where the top and side views of the paths are depicted in the left panel for two diffusion directions (indicated by red and black arrows respectively). The structural models are given for the initial, transition and final states, respectively. Brown, white, gray, blue, and pink balls represent C, H, N, Cu substrate, and Cu adatoms, respectively. Source data are provided as a Source data file.

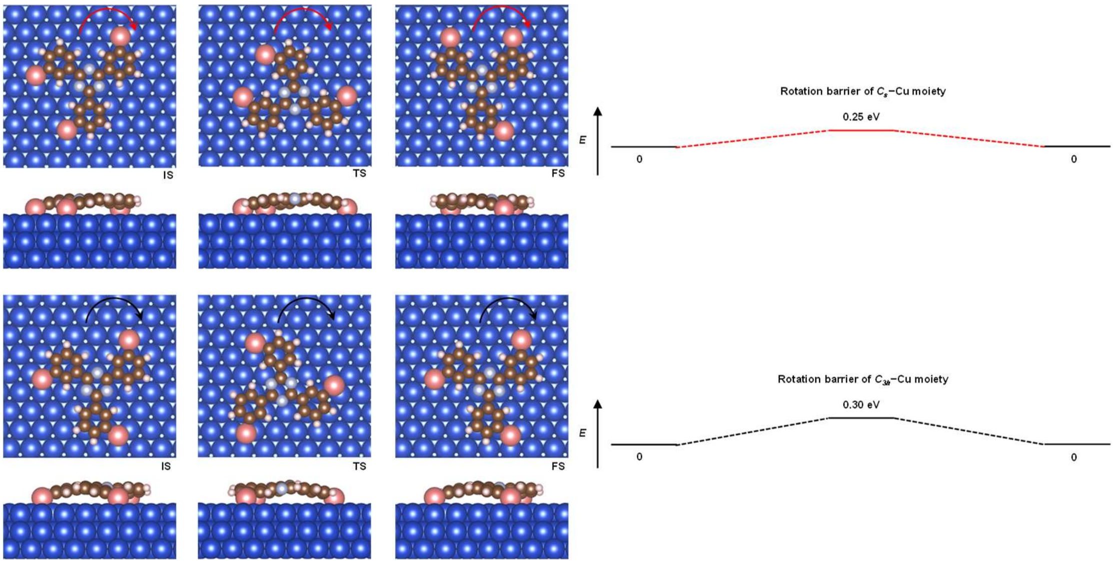

Supplementary Figure 12. Energy barriers for rotations barriers of C_s−Cu and C_{3h}−Cu moieties, respectively. DFT-calculated energy diagrams for rotation processes of the C_s−Cu (upper panel) and C_{3h}−Cu (lower panel) moieties on Cu(111). The structural models are given for the initial, transition and final states, respectively. Brown, white, gray, blue, and pink balls represent C, H, N, Cu substrate, and Cu adatoms, respectively. Source data are provided as a Source data file.

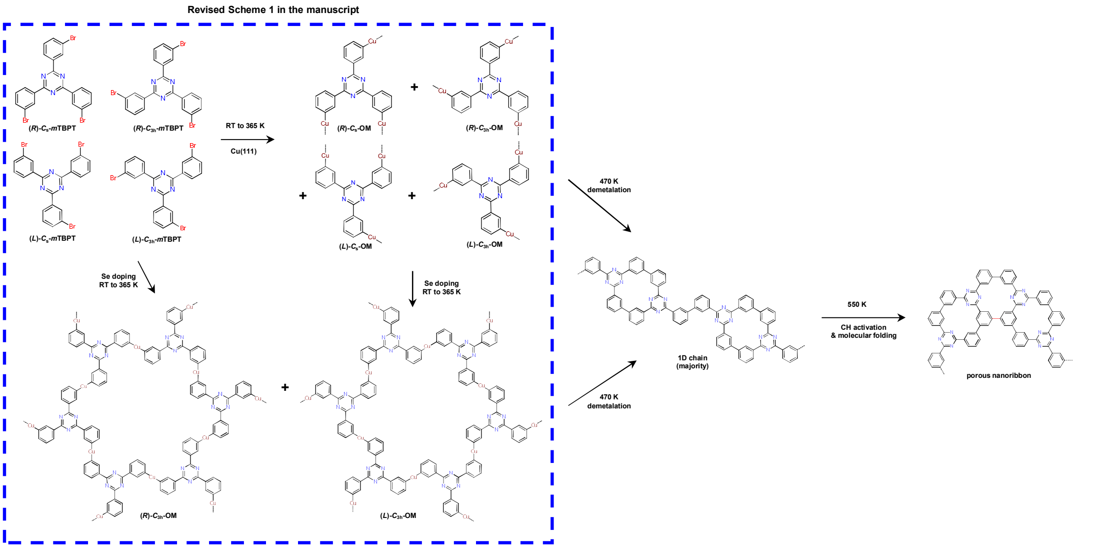

Supplementary Figure 13. Scheme of *m*TBPT reaction pathway. The blue dashed rectangle framed the revised Scheme 1 in the manuscript. No matter random (without Se doping) or ordered (with Se doping) organometallics went through demetallation to form 1D covalent interlinked chains (470 K), and finally C−H activation was triggered to form porous N-doped nanoribbon with further annealing (550 K).

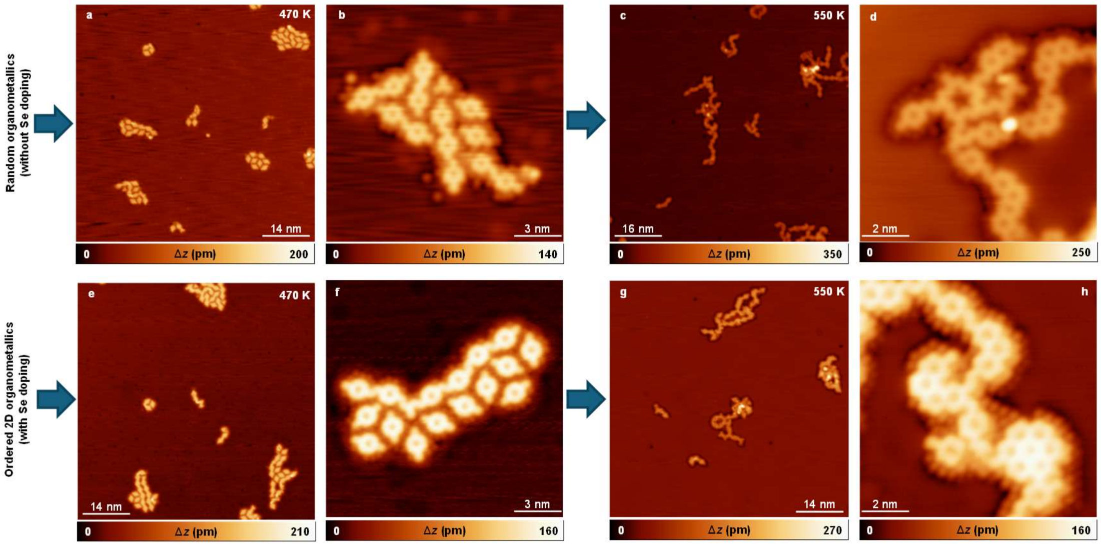

Supplementary Figure 14. STM images after further annealing from random (without Se doping) or ordered (with Se doping) organometallics. (a) Large-scale and (b) close-up STM images showing 1D covalent interlinked chains (majority) after annealing at 470 K from the random organometallics (without Se doing). (c) Large-scale and (d) close-up STM images showing the N-doped porous graphene nanoribbon after further annealing at 550 K (without Se doing). (e) Large-scale and (f) close-up STM images showing 1D covalent interlinked chains (majority) after annealing at 470 K from the ordered 2D organometallics (with Se doing). (g) Large-scale and (h) close-up STM images showing the N-doped porous graphene nanoribbon after further annealing at 550 K (with Se doing). STM parameters: constant current, (a-c, g) *U*

= -1 V, I = 100 pA; (d) U = -500 mV, I = 100 pA; (e) U = 1 V, I = 100 pA; (f) U = 500 mV, I = 50 pA; (d) U = -500 mV, I = 50 pA.

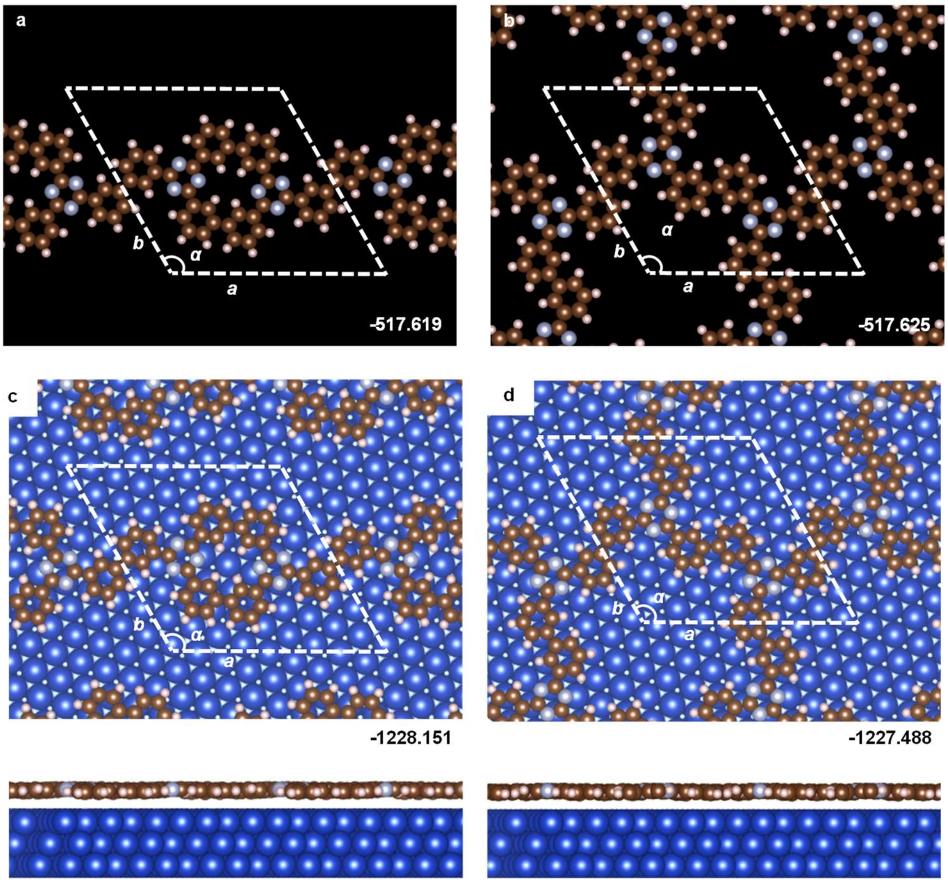

Supplementary Figure 15. DFT calculations on the covalent interlinked 1D chain and 2D network. DFT relaxed models of covalent interlinked (a) 1D chain and (b) 2D network in the gas phase. DFT relaxed models of covalent interlinked (c) 1D chain and (d) 2D network on Cu(111). The calculated energies were also indicated in the unit of eV. Brown, white, gray, and blue balls represent C, H, N, and Cu substrate, respectively. Source data are provided as a Source data file.
